# Degenerative Cervical Myelopathy: History, Physical Examination, and Diagnosis

**DOI:** 10.3390/jcm13237139

**Published:** 2024-11-25

**Authors:** Mariah Balmaceno-Criss, Manjot Singh, Mohammad Daher, Rachelle Buchbinder, Bassel G. Diebo, Alan H. Daniels

**Affiliations:** 1 Department of Orthopedics, The Warren Alpert Medical School of Brown University, Providence, RI 02903, USA; mariah_bc@brown.edu (M.B.-C.); manjot_singh@brown.edu (M.S.); mohdaher06@gmail.com (M.D.); dr.basseldiebo@gmail.com (B.G.D.); 2 Musculoskeletal Health and Wiser Health Care Units, School of Public Health and Preventive Medicine, Monash University, Melbourne, VIC 3004, Australia; rachelle.buchbinder@monash.edu

**Keywords:** degenerative cervical myelopathy, clinical presentation, physical examination, diagnosis, management, referral

## Abstract

**Background**: Degenerative cervical myelopathy is a progressive neurological disorder that is commonly encountered in clinical practice and its incidence is expected to increase alongside the aging population. Given the importance of early and accurate diagnosis in this patient population, this narrative review aims to provide a repository of up-to-date information regarding pertinent patient history, physical exam findings, and potential alternate diagnoses. **Methods**: The PubMed database was queried for publications from 1 January 2019 to 19 March 2024. The search terms utilized are as follows: cervical myelopathy”, “cervical spondylotic myelopathy”, “degenerative cervical myelopathy”, “epidemiology”, “prevalence”, “incidence”, “etiology”, “diagnosis”, “differential”, “symptoms”, “clinical presentation”, and “atypical symptoms”. The resultant articles were reviewed for relevance and redundancy and are presented within the following categories: Natural History, Epidemiology, Clinical Presentation, Diagnosis, and Management. **Results**: Myelopathy patients often present with subtle and non-specific symptoms such as sleep disturbances, increased falls, and difficulty driving, which can lead to underdiagnosis and misdiagnosis. Failing to diagnose degenerative cervical myelopathy in a timely manner can result in progressive and irreparable neurological damage. Although many nonoperative treatment modalities are available, surgical decompression is ultimately recommended in most cases to limit further deterioration in neurological function and optimize long-term patient outcomes. **Conclusions**: A thorough clinical history and physical examination remain the most important diagnostic tools to avoid misdiagnosis and implement early treatment in this patient population.

## 1. Introduction

Degenerative cervical myelopathy (DCM) is a general term that refers to symptomatic spinal cord compression secondary to a range of degenerative changes to the cervical spine. In recent studies, DCM has been associated with greater physical and mental disability than other common pathologies, including myocardial infarction, cancer, and adult spinal deformity [1]. As patients with DCM have a high risk for progression and neurologic deterioration, it is important to recognize and manage this disorder in a timely manner [2]. Unfortunately, DCM is often underdiagnosed or misdiagnosed due to its variable clinical presentation and disease severity, as well as common overlap in symptoms with other neurological and musculoskeletal disorders [3,4]. Failure to adequately diagnose and manage DCM can result in severe neurologic deterioration and significantly impact patients’ quality of life. Timely diagnosis of DCM requires knowledge of its natural history, clinical presentation, and diagnostic modalities. In addition, it requires a thorough understanding of the proper timing for referral to specialists. As such, the aim of this review is to identify and summarize the literature on cervical myelopathy with a focus on patient history, physical examination, and differential diagnosis in order to improve early recognition of the disease by primary care providers. To be comprehensive, this review will also touch on nonoperative and operative management of the disease.

## 2. Search Strategy

For this narrative review, we searched the PubMed database for relevant publications from 1 January 2019 to 19 March 2024, using the following search terms: “cervical myelopathy”, “cervical spondylotic myelopathy”, “degenerative cervical myelopathy”, “epidemiology”, “prevalence”, “incidence”, “etiology”, “diagnosis”, “differential”, “symptoms”, “clinical presentation”, and “atypical symptoms”. The sources cited in the articles generated from the query were also reviewed. Given the limited epidemiological data available, source time range was extended to also include articles published from 2013 onward.

## 3. Natural History

DCM is a progressive condition characterized by a gradual stepwise decline in functional status, with multiple phases of neurologic decline followed by periods of stabilization [5]. Initial symptoms may be subtle, such as sleep disturbances, increased falls, and difficulty driving, and are often erroneously attributed to the normal aging process, thus delaying clinical diagnosis [6,7,8]. However, a substantial proportion of DCM patients, ranging from 57% to 95%, experience some decline in neurological status and only a small fraction achieve prolonged remission after symptom onset [9,10]. Identified predictors of neurologic deterioration include duration of symptoms, comorbid congenital stenosis, T2-weighted hyperintensity in the cervical spinal cord on MRI, ossification of the posterior longitudinal ligament, and poorer preoperative neurologic function [11,12,13,14,15]. Without timely intervention, patients may ultimately develop severe weakness or paralysis.

## 4. Epidemiology

### 4.1. Incidence and Prevalence

With an increasing and aging population, as well as reduced mortality from communicable diseases, the burden of DCM is expected to rise. However, there are few data on the true prevalence and incidence of DCM worldwide [16,17]. Early studies estimated a prevalence of 3.5 per 1000 cases and reported DCM as the most common cause of non-traumatic paraparesis and tetraparesis in adults [18,19]. Using data from the National Health Insurance Research Database from 1998 to 2009, Wu et al. reported a DCM-related hospitalization incidence of 4.04 per 100,000 person-years in Taiwan [20]. Nouri et al. subsequently estimated the incidence to be 41 per million people in North America [21]. Most recently, Smith et al. reported a pooled prevalence of DCM 2.3% (95% CI 1.4 to 3.1), based upon three studies including 1202 healthy people (mean age 45–66 years, studies from Canada, Japan, and the Czech Republic; low-quality evidence) [22].

### 4.2. Age and Sex Predominance

Degenerative pathologies increase with age. Matsumoto et al., for instance, previously observed that disc degeneration among men and women increased from 17% and 12% in their twenties to 86% and 89% in their sixties, respectively [23]. The age-related prevalence of DCM also increases in a similar fashion, with a peak prevalence of 0.42% in people aged 50–54 years [3,22]. More broadly, studies suggest that people aged 45–64 years are at an increased risk of DCM and subsequent spinal fusions [3,18,24]. The prevalence is generally higher in males, with a male-to-female ratio of 2.7:1 [20,25].

### 4.3. Risk Factors

**Figure 1:**
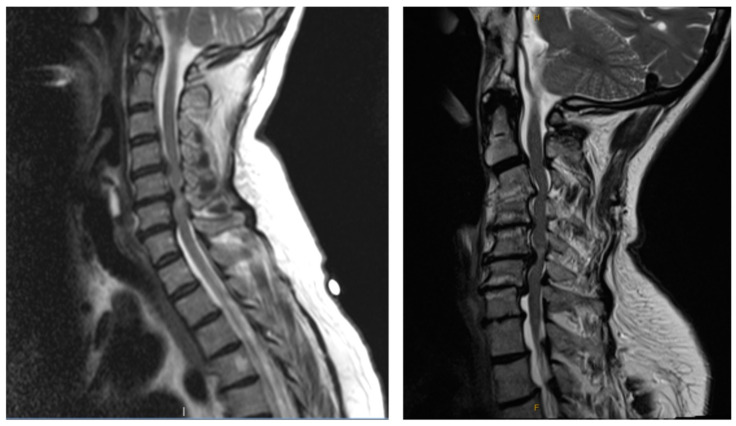
T2-weighted MRI demonstrating a patient with congenital stenosis and multi-level stenosis from disc herniations (**left**) and a patient with congenital stenosis and multi-level stenosis from disc degeneration and kyphotic deformity (**right**).

There are multiple risk factors that may predispose patients to DCM. Demographic factors, including increasing age, male sex, and relative socioeconomic deprivation, have been independently associated with DCM [26]. Congenital anomalies, such as congenital cervical stenosis (i.e., anteroposterior canal diameter < 10 mm) or abnormalities of the atlas or axis (e.g., unilateral facet cyst, os odontoideum), can accelerate degenerative compression of the cervical spinal cord and result in earlier presentation of symptoms (Figure 1) [27,28,29,30,31,32,33]. Metabolic syndrome, such as obesity, diabetes, and hyperlipidemia, can promote ossification of the posterior longitudinal ligament and contribute to stress-related ischemic injury of the cervical spinal cord [34,35,36]. Traumatic injuries of the cervical spine can result in osteophytic or heterotrophic bone formation and direct compression of the spinal cord [37,38]. Finally, although these have been less extensively studied, gout, pseudo-gout, and Tourette’s have also been associated with DCM [39,40].

## 5. Clinical Presentation

DCM is a challenging clinical diagnosis as the presenting symptoms and signs vary depending on the location and degree of spinal cord compression. Moreover, compression may involve multiple cervical spinal levels, further complicating the clinical picture. Symptoms often associated with DCM include neck or upper extremity pain, paresthesia, and muscle weakness [41,42,43,44]. When combined, sensory disturbance and weakness can contribute to hand clumsiness and difficulty performing tasks involving fine motor skills, such as buttoning a shirt, using keys, or turning doorknobs. Symptoms can also extend into the lower extremities and contribute to gait instability, which is notably wide-based and ataxic on clinical examination. Bowel and bladder dysfunction may also be reported by DCM patients, often including urinary incontinence and constipation, though a wide variety of bowel and bladder symptoms have been documented [45,46].

### Clinical Variability

Given the heterogeneity of clinical presentation, multiple studies have aimed to quantify the frequency and distribution of presenting symptoms. A recent scoping review that quantified weight averages for symptom frequency based upon 58 studies including cohorts of patients with DCM (N = not provided) and 3 diagnostic accuracy studies (N ranging from 33 to 100) identified hand numbness and paresthesia as the most frequent and sensitive symptoms (Table 1) [46].

Recognizing atypical clinical presentations of DCM is also imperative to avoid missed and late diagnoses. While most patients present with upper extremity symptoms, one retrospective case series of 982 surgically treated patients found that 1.2% had no upper extremity symptoms at presentation [47]. All of these patients had difficulty ambulating and over half (7/12) had objective lower extremity weakness. Atypical symptoms, such as abdominal pain, tremors, headache, vertigo, and tinnitus have also been reported in patients with DCM [48,49,50].

## 6. Diagnosis

### 6.1. Patient History

When assessing a patient with suspected DCM, a series of open-ended and targeted questions are crucial to eliciting appropriate pertinent history. Open-ended questions should aim to assess symptom onset, progression, and exacerbating factors. Symptom onset is often insidious and slowly progressive in cervical myelopathy while a more acute onset in the absence of acute trauma may support an alternative diagnosis. Targeted questions should aim to assess the presence of myelopathy-specific symptoms [51]. Upper or lower extremity pain and paresthesia are often the presenting symptom. Diminished fine motor control may be assessed by determining whether the patient has difficulty buttoning a shirt, putting on jewelry, or has noticed changes in their handwriting. Weakness of the intrinsic hand muscles can be assessed by asking whether the patient has difficulty opening jars, holding a pencil, or often drops objects. Gait disturbance can be ascertained through questions about whether patients feel unsteady when walking, if they have ever lost their balance, if they require assistive devices during ambulation (handrails, canes, etc.), and if they have any history of falls. Bowel and bladder dysfunction can be obtained by inquiring about episodes of hesitancy, urgency, or incontinence.

### 6.2. Physical Examination

A targeted yet thorough neurologic examination should be performed in all patients with suspected DCM [52]. Motor assessment of both the upper and lower extremities with particular attention to muscle tone and grip strength should be performed. Patients with DCM often have motor weakness and atrophy of the intrinsic hand muscles [53]. Evaluating pinprick, temperature, and vibration sense as well as proprioception allows for assessment of the integrity of the spinothalamic tract and the dorsal column–medial lemniscus pathway. While DCM patients are more likely to present with proprioceptive dysfunction, depending on the degree of compression, patients may also have disrupted temperature and pain sensation. Additionally, reflex testing, including Babinski and clonus, is an important component of the examination, as many patients with DCM exhibit hyperreflexia [53,54]. Finally, assessing gait with tandem walking is pertinent to assess for stride length, base width, and any loss of coordination. Gait dysfunction in DCM is often described as broad-based [55]. Examination of cognitive function and mental status is also worthwhile as part of fall risk assessment and to determine the appropriateness of different treatment options. Additional examination, for example, of the cranial nerves, may also be indicated to rule out alternative diagnoses.

Outside of the standard neurologic examination, special tests may be performed to further assess patients. The Romberg test is a qualitative test to assess proprioception and postural stability; the test is abnormal in DCM patients with dysfunction of the dorsal column–medial lemniscus pathway. The Romberg test is performed by having the patient stand with both feet together with their hands held next to their body first with their eyes open and then closed [52]. A positive test is signified by swaying, loss of balance, or falling when the patient’s eyes are closed. Recent studies have explored modification of the Romberg test with force plate analysis for a more quantitative evaluation of the severity of dysfunction, finding greater sway area and speed in patients with DCM compared to controls [56,57].

Hoffman’s sign, elicited by flicking the dorsal aspect of the third finger’s distal phalanx downward, is positive if involuntary flexion of the ipsilateral thumb or index finger is observed and is a sign of upper motor neuron dysfunction [51,58]. Another special test used to evaluate potential DCM is the inverted supinator sign/inverted radial reflex, which when positive signifies upper motor neuron dysfunction. The inverted supinator sign/inverted radial reflex is elicited by tapping the attachment of the brachioradialis tendon; if the only response is hyperactive finger flexion, the test is positive [59].

A systematic review that synthesized the available evidence for the diagnostic accuracy of various clinical signs found that based upon 11 included studies (N ranging from 45 to 7629), Tromner’s sign and generalized hyperreflexia were the most sensitive clinical signs and Babinski, Tromner’s sign, clonus, and the inverted supinator sign were the most specific (Table 2) [60]. The combination of positive finger flexion, Hoffmans, and Babinski signs had a sensitivity of 91.7%, specificity of 87.5%, positive predictive value of 95.7%, and negative predictive value of 77.8% in detecting spinal cord compression in another study by Tejus et al. that included 32 cases and 302 healthy controls [61]. While further studies are needed to confirm these findings, the main conclusion to be drawn from the current literature is that multiple positive signs should increase clinical suspicion for DCM and warrant further diagnostic work up.

### 6.3. Advanced Imaging and Diagnostic Tests

The first diagnostic imaging test should be plain radiographs of the cervical spine. Standard AP and lateral cervical radiographs can be used to assess the extent of cervical degeneration, notably the loss of disc height, end plate abnormalities, and presence of osteophytes. Lateral flexion and extension radiographs allow for the identification of hypermobility and instability.

**Figure 2:**
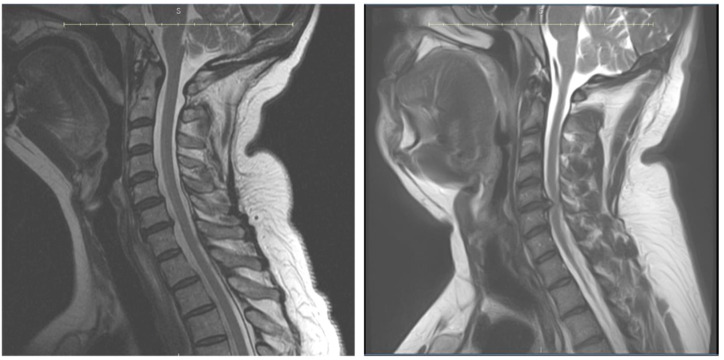
T2-weighted MRI demonstrating a patent cervical canal (**left**) and single-level stenosis from a large C5/6-disc herniation (**right**).

The diagnostic imaging test of choice for DCM is MRI. MRI can be used to identify the degree of canal stenosis, visualize spinal cord compression, and signal cord changes such as myelomalacia (Figure 2) [62,63,64]. Although spinal cord compression has high sensitivity for DCM, cord compression has also been identified in MRI studies of asymptomatic populations; therefore, MRI findings should always be correlated with clinical features [22,34,65,66,67]. Conversely, T2-weighted hyperintensity has been demonstrated to have high specificity for DSM, but it is not always present and it is a poor predictor of patient outcome, limiting its diagnostic value [65,68,69]. Emerging research utilizing microstructural and functional MRI techniques have shown promising diagnostic potential but require further evidence of benefit to support widespread use [70,71,72]. MRI is often necessary to rule out alternative diagnoses, such as compression from a structural lesion.

In some cases, electrodiagnostic studies, including nerve conduction studies (NCS), electromyography (EMG), somatosensory evoked potentials (SSEPs), and motor evoked potentials (MEPs), may be useful adjunct tests. While nerve condition and EMG studies are often normal in people with DCM, these studies are useful to identify cases of myeloradiculopathy or rule out alternative diagnoses such as peripheral neuropathies or ALS [73]. Abnormalities in the dorsal column–medial lemniscus pathway show somatosensory evoked potentials with decreased amplitude, increased latency, and increased waveform dispersion, but application of SSEPs as a diagnostic tool is limited given poor sensitivity [70,74,75,76,77]. MEPs can be used alongside nerve conduction studies to determine prolonged central motor conduction time, which has been useful in identifying ALS, MS, and DCM, and are generally more sensitive and specific than SSEPs [70,76,78,79]. Similar to neuroimaging, the application of electrodiagnostic testing in DCM is rapidly advancing. A recent study by Pilato et al. explored the prognostic efficacy of combined use of neuroimaging and electrodiagnostic studies [80]. In their investigation, Pilato et al. developed a comprehensive scoring system accounting for clinical, electrodiagnostic, and neuroimaging findings to predict surgical prognosis and demonstrated the comprehensive score was more accurate compared to each modality alone [80].

### 6.4. Classification

The most frequently used assessments for the severity of disability in patients with DCM are the modified Japanese Orthopaedic Association Scale (mJOA) and the Nurick grade [81,82]. The multidimensional mJOA scale includes upper and lower extremity motor function, upper extremity sensation, and urinary symptoms; it is scored from 0 to 18 points, with a lower score representing greater disability [83]. An mJOA score between 15 and 17, 12 and 14, and 0 and 11 indicates mild, moderate, and severe myelopathy, respectively [83,84]. The Nurick grade is a five-point scoring system that assesses the stepwise neurologic decline in ambulatory ability [55].

While the above classification systems are of value in determining disability severity and informing treatment, they are not typically used for screening potential DCM patients. More recently, Barkoh et al. proposed the DOWN Questionnaire, a four-item screening test covering symptoms of hand clumsiness, imbalance, and upper extremity motor and sensory deficits [85]. In their study, positive responses to three or more questions had high sensitivity and moderate agreement with the diagnosis of myelopathy based upon history, physical examination, and review of advanced imaging by a spinal surgeon. These data indicate that the DOWN questionnaire may be a useful screening tool in the clinic, but these results require further validation in other settings.

### 6.5. Differential Diagnoses

There are several pathologies that share similar characteristics to degenerative cervical myelopathy. A list of these conditions, as well as their clinical presentation, is provided in Table 3.

## 7. Management

The management of DCM depends upon the severity of the disease at clinical presentation [86]. In mild disease, reassurance, counseling on the risk of progression, education on ‘red flag’ symptoms that should prompt early review (e.g., new neck pain, insidious onset, neurologic deterioration), and regular clinical follow-up should be provided. Nonoperative modalities, including physical therapy, simple analgesia, and orthoses may be considered to alleviate symptoms such as neck pain. However, there is a paucity of trial evidence to support nonoperative treatments or to suggest that nonoperative treatment can halt or reverse DCM progression. Regular clinical review, including physical examination, is recommended to detect signs of disease progression. Neurologic deterioration and initial presentations with moderate or severe disease should prompt consideration of operative modalities to prevent further progression. There is also limited trial evidence about surgical interventions or to recommend one operative approach over another.

### 7.1. Nonoperative Management

While early surgical intervention is generally recommended due to the progressive nature of the disease, nonoperative modalities may be offered to patients with mild DCM guided by their specific symptoms. In the absence of any placebo-controlled trials in patients with DCM, patients can be informed that high certainty evidence of benefit is lacking across all nonoperative treatments. For pain, a trial of simple analgesics such as paracetamol and non-steroidal anti-inflammatory drugs may be offered depending upon the presence of comorbidities. A small open randomized pilot trial (N = 39) assessed the effect of adding pregabalin (150 mg daily for first week, 300 mg/day for second week then 600 mg daily for 6 weeks) to opioids (5 mg oxycodone three times a day) for 8 weeks and reported small benefits in the Leeds assessment of neuropathic symptoms and signs (LANSS) and neck and arm pain at 4 but not 8 weeks [87]. Between-group differences however may be explained by bias, and further high-quality trials are needed before gabapentinoids can be recommended. While physical therapy has been employed to reduce compressive forces on the cervical spine in patients with cervical radiculopathy, evidence for its effectiveness in patients with DCM is scarce. Finally, psychological management, with careful consideration of patients’ demographic and clinical characteristics, can also be employed to manage chronic pain [88,89,90]. While specific research on DCM is limited, evidence from related conditions highlights the potential benefits of integrating positive psychology to enhance coping and overall quality of life.

Active research is currently exploring therapeutic options to improve neurologic recovery in patients with DCM. Mesenchymal stem cells and therapeutic protein injections may have promise, although are still in the early phases of development [91,92]. Repetitive transcranial magnetic stimulation has also been proposed and may have some role in enhancing neurologic recovery [93]. Several placebo-controlled trials have also explored the efficacy of neuroprotective medications for promoting neurologic recovery [94,95,96,97,98,99]. One placebo-controlled trial of Cerebrolysin, a mixture of multimodal neuropeptides believed to have neurotrophic, neuroprotective and neuroregenerative effects (IM injection given 5 days per week for 4 weeks), in 192 participants with DCM who were surgical candidates but declined surgery reported significant improvements in myopathy up to 6 months, favoring the active drug [97]. Two additional placebo-controlled trials that assessed the value of Cerebrolysin given daily for either 10 or 21 days also reported favorable results although the clinical importance of between-group differences were less apparent [98,99]. One multicenter placebo-controlled trial (n = 290 participants) did not find any benefits in functional recovery of riluzole, a benzothiazole that has been used to treat amyotrophic lateral sclerosis, as an adjunct to decompressive surgery in people with moderate to severe DSM [96]. Another placebo-controlled trial of ibudilast, a phosphodiesterase 3/phosphodiesterase 4 inhibitor, as an adjunct to decompressive surgery is also underway [95].

### 7.2. When to Refer

Primary care clinicians should have a low threshold for referral to a specialist if DCM is suspected. Early referral in the elderly population is especially vital as advanced patient age, greater duration of symptoms, and more severe preoperative symptoms are the greatest predictors of poorer outcomes [100]. If possible, plain radiographs and MRI of the cervical spine should be obtained prior to any referrals. For patients with a confirmed diagnosis of DCM, immediate referral to a spine surgeon in the field of orthopedics or neurosurgery for consideration of surgical management is indicated. If the initial workup is negative, the patient may be referred to a neurologist or rheumatologist for further evaluation.

### 7.3. Operative Management

For the symptomatic patient, surgical decompression is indicated to alleviate mechanical compression of the cervical spinal cord. The ideal surgical candidate is a younger individual with few comorbidities and absent radiographic myelopathy signs because these patients have the greatest potential for improvement in functional outcomes [83,101]. However, surgery is recommended for all DCM patients irrespective of preoperative demographic and radiographic variables, especially among those with higher disease severity and worse functional status, to slow neurologic decline [102,103]. A patient’s specific risk for neurologic decline can be determined using a formula generated by Sarraj et al. and understanding their risk of decline can assist with shared decision making between the patient and provider regarding whether surgery would provide a quality of life benefit to the patient [104]. Surgical options for the management of DCM include anterior (e.g., anterior cervical discectomy and fusion, cervical disc arthroplasty), posterior (e.g., laminoplasty, laminectomy with fusion, and skip laminectomy), and combined (anterior cervical discectomy and fusion with posterior laminectomy and fusion) approach-based interventions [55].

Despite various trials assessing outcomes associated with different surgical approaches, there is a notable absence of placebo-controlled trials for the surgical treatment of DCM and only two randomized controlled trials that have compared surgical intervention to nonoperative treatment [105,106]. Both trials reported no between-group differences up to 3 years postoperatively, but their findings are limited by methodologic weaknesses including potential selection bias and underpowered sample sizes. While requiring confirmation in high-quality trials, single-arm studies have suggested that surgery may improve functional status, such as increased hand strength and dexterity, and quality of life, with the degree of improvement depending on the age of the patient [45,107,108,109]. The risks of surgery include surgical complications (e.g., postoperative pain, neurologic injury, infection), persistence of symptoms, and postoperative deterioration of symptoms [109,110,111,112,113,114].

Patients contemplating surgery should be counseled that they may not note any improvement in functional outcomes, though some studies indicate that the vast majority of the patients do experience some improvement, and nearly 37% may improve one grade in myelopathy severity [55]. Newer studies have examined the use of machine learning algorithms and novel surgical interventions, such as full endoscopic spine surgery, to predict and improve postoperative outcomes [115,116,117]. However, a multidisciplinary approach involving close collaboration between the patient, primary care physician, and spine surgeon is ultimately needed to optimize outcomes in patients undergoing DCM surgery [118,119].

## 8. Limitations

This narrative review on DCM has several potential limitations. While a thorough assessment of the current literature was performed, this type of review lacks a systematic methodology to identify and summarize the data presented by all relevant articles. In addition, the quality of the articles described here was not reviewed and may add potential bias. Finally, there may be inherent risk of subjectivity in the selection of articles and interpretation of results. Nevertheless, the literature is clear on the significant impact of DCM on patients and on the need to adequately identify and manage this condition in a timely manner. This review may thus help guide physicians on our current understanding of DCM.

## 9. Conclusions

DCM is a progressive degenerative disease that results from cervical spinal cord compression. Its incidence and prevalence are expected to rise with the aging population. Despite its tremendous clinical impact, the diagnosis of DCM is often elusive due to variable initial presentation and disease severity. Clinical diagnosis relies on comprehensive assessment encompassing patient history, physical examination, and consideration of differential diagnoses, with advanced imaging and diagnostic tests playing crucial roles. Treatment spans from initial nonoperative modalities for mild disease to surgical decompression for those who progress and for moderate to severe disease. Ultimately, diagnosis and management of patients with DCM requires interdisciplinary collaboration, and implementation of evidence-based diagnostic and therapeutic strategies to optimize patient care and quality of life, ideally supported by high-quality randomized controlled trials.

## Figures and Tables

**Table 1:** Weighted sensitivity of symptoms from a scoping review by Jiang et al., 2023 [46].

Symptom	Frequency	Sensitivity
	% Range	% (95% CI)
Hand numbness	21 to 89	82 (80 to 85)
Hand paresthesia	24 to 93	79 (68 to 87)
Upper extremity numbness	4 to 96	69 (66 to 72)
Hand clumsiness	26 to 90	69 (67 to 72)
Upper extremity weakness	4 to 92	58 (55 to 60)
Upper extremity paresthesia	29 to 70	57 (54 to 60)
Neck/shoulder pain	9 to 100	51 (49 to 53)
Upper limb pain	10 to 54	43 (40 to 46)
Fine motor disturbance	22 to 71	29 (25 to 33)
Hand weakness	4 to 18	10 (3 to 24)
Gait dysfunction	10 to 100	72 (70 to 74)
Lower extremity numbness	17 to 91	61 (57 to 66)
Lower extremity weakness	3 to 81	54 (51 to 57)
Gait imbalance	4 to 25	23 (19 to 27)
Back pain	9 to 22	19 (14 to 27)
Unspecified paresthesia	85 to 92	86 (82 to 90)
Axial pain	19 to 100	41 (35 to 46)
Radicular pain	7 to 96	39 (35 to 42)

**Table 2:** Weighted sensitivity and specificity of clinical signs from a systematic review by Jiang et al., 2023 [60].

Sign	Frequency	Sensitivity	Specificity
	% Range	% (95% CI)	% (95% CI)
Tromner’s	NR	94 (86 to 98)	93 (81 to 99)
Hyperreflexia (generalized)	33 to 100	72 (55 to 85)	43 (27 to 61)
Hoffman’s	21 to 100	58 (53 to 63)	72 (68 to 76)
Motor impairment	9 to 100	54 (53 to 56)	64 (63 to 65)
Sensory impairment	19 to 100	48 (41 to 55)	66 (56 to 76)
Inverted supinator	NR	41 (34 to 48)	93 (89 to 96)
Babinski’s	11 to 100	17 (11 to 23)	99 (97 to 100)
Clonus	3 to 73	9 (5 to 15)	99 (96 to 100)

Abbreviation: NR = not reported.

**Table 3:** Differential diagnosis for degenerative cervical myelopathy and pertinent clinical history questions.

Differential Diagnoses	Clinical Presentation	Questions for Patient History
Amyotrophic lateral sclerosis	Muscle weakness, stiffness, spasticity, hyperactive reflexes, muscle atrophy, clumsiness, bulbar symptoms, preservation of bowel and bladder function, and absence of sensory symptoms.	Has anyone noticed a change in your voice? Do you have difficulty swallowing? Do you feel jumpy (can signify fasciculations)?
Multiple sclerosis	Sensory disturbances, motor weakness, optic neuritis, diplopia, hearing loss, fatigue, impaired coordination, bowel and bladder dysfunction.	Have you had prior episodes of impaired or loss of vision? Ask about family history of autoimmune disorders.
Normal pressure hydrocephalus	Subacute or chronic gait disturbance, bladder detrusor overactivity, cognitive slowing (often a late finding).	
Syringomyelia	Cape-like distribution of motor weakness and loss of pain and temperature, gait dysfunction, headaches, and dizziness.	Ask about history of neck trauma (i.e., motor vehicle accidents). Do you have a history of headaches?
Hereditary spastic paraparesis	Spastic paraparesis, gait disturbance progressive, hyperreflexia, urinary dysfunction, and minor loss of lower extremity vibratory sense.	Ask about history of similar symptoms in family members.
Metabolic myelopathy	Gradual onset, distal symmetric sensory loss of vibration and proprioception, gait dysfunction, and weakness (late finding).	Ask about a history of gastric surgeries. Ask about history of veganism and alcohol consumption.
Peripheral neuropathy (i.e., carpal tunnel)	Pain and sensory dysfunction in a dermatomal distribution with atrophy as a late finding.	Are your symptoms worse at night? Does shaking your hands help relieve your symptoms?
Structural lesion	Signs and symptoms based on location of compression.	Have you experienced any systemic symptoms (i.e., fever, weight loss)?

## Data Availability

Data are contained within the article.
